# Defining competence profiles in obstetrics and gynecology using the modified requirement tracking questionnaire

**DOI:** 10.1186/s12909-025-06806-7

**Published:** 2025-02-07

**Authors:** Baptiste Tarasi, Viktor Oubaid, David Baud

**Affiliations:** 1 Materno-Fetal and Obstetric Research Unit, Woman-Mother-Child Department, University Hospital of Lausanne, CHUV, Lausanne, 1011 Switzerland; 2 German Aerospace Centre (DLR), 22335 Hamburg, Germany

**Keywords:** Competence, Gynecology and obstetrics, Residency selection, Postgraduate medical education

## Abstract

**Background:**

Data about the competencies needed for physicians in obstetrics and gynecology (O&G) is currently insufficient. The aim of this study is to define the competence profile needed in this sector for daily professional activity, in order to account for these criteria in future recruitment.

**Methods:**

The modified requirement-tracking questionnaire (R-track) was sent to 307 physicians working in the field of O&G with different training levels and practice locations. The R-track is designed to assess professional competence profiles and contains 66 items covering the following eight competence areas: “Mental abilities”, “Social sensibility”, “Psychomotor and multitasking abilities”, “Solutions orientation”, “Social interactive competences”, “Personality traits”, “Verbal competences” and “Resistance capacity”. The mean scores of single items and competence areas were calculated. Results were compared between gender, training level, and place of practice.

**Results:**

The participation rate was 65.5%, with 201 physicians returning the questionnaire. In this sample, 50.2% of them were in training and 49.8% were practicing O&G specialists. The proportion of physicians working in a hospital setting was 64.7% while 30.3% worked in private practice. The competence areas “Social sensibility” and “Psychomotor & multitasking abilities” appear to be the most important for daily professional activity. At the item level, “Stress resistance”, followed by “Workload management” and “Tactfulness” were considered as the most valuable characteristics. Differences between gender, level of training, and place of practice were not significant.

**Conclusion:**

The identified competence profile could serve as a basis for developing a new method of O&G residency selection. In addition, such a profile could help medical students to decide on a professional specialization at a very early stage by comparing their personal competence profile with the one in the field or with their mentors.

**Supplementary Information:**

The online version contains supplementary material available at 10.1186/s12909-025-06806-7.

## Introduction

The selection of medical graduates for postgraduate training can be carried out using different methods, including standardized tests, academic performance and medical school grades, internship reports, letters of recommendation, extracurricular activities, or personal interviews [1–4].

There is no consensus, however, on the superiority of a selection system for identifying optimal candidates for a training program [5]. Although some studies have shown stronger associations between standardized test results or medical school grades and physician performance during their residency compared with the other selection methods [2, 6], a direct correlation with these results is frequently not found [5, 7–8]. A focus on academic performance also ignores the findings from other high-security industries, which have proven the importance of personality and social skills in increasing safety and preventing accidents. For example, due to evidence from scientific research, regular Crew Resource Management (CRM) training has been incorporated into the professional commercial pilot license for many years and CRM assessment is part of every mandatory official pilot check [9].

As medical specialties are diverse and varied, personalizing a selection process is essential to reflect the values and needs of a specific program [5]. Indeed, the required competences of a resident differ from one medical specialty to another [10–11]. Although distinctive personality traits can be associated with physician specialty choices after medical school [12–13], the compatibility between a physician’s competences and those required by his or her specialty is subsequently associated with well-being at work and professional effectiveness [7, 14]. However, there is insufficient literature on how key skills are considered in recruitment processes and their impact on candidate selection.

O&G is a medical specialty that focuses on the health of the female reproductive system. It encompasses prenatal care, childbirth, postnatal care, and the diagnosis and treatment of gynecological disorders throughout a woman’s life, from adolescence through menopause and beyond. It requires a wide range of both medical and surgical skills, but data regarding required competences for daily professional activity are limited.

Our study seeks to define the competence profile in O&G with the aim of subsequently better aligning residency selection and training with the demands of the specialty. By doing so, we hope to contribute to a more structured and evidence-based recruitment process that emphasizes competencies directly related to successful practice in O&G.

## Methods

To identify the competence profile required in O&G, an online questionnaire was sent via a mailing list to all 307 physicians working in the field of O&G in the French-speaking part of Switzerland, excluding those practicing in the canton of Geneva. The mailing list included both physicians in residency programs and trained specialists working in hospitals or private practices. Participation was voluntary and took place over a two-month period, from December 1, 2021, to January 31, 2022.

The questionnaire included the following demographic information: age, gender, level of training, place of practice, and years of professional experience, in addition to the modified requirement-tracking questionnaire (R-track) (supplement 1). The R-Track questionnaire, inspired by the Fleishman Job Analysis Survey [15], was originally designed to assess necessary skills and abilities required to successfully fulfill professional tasks in the field of aviation and aerospace, and has since been developed for other occupational groups, such as physicians.

The decision to use the R-Track questionnaire was based on two main reasons. First, essential areas such as personality traits and social-interactive competencies are either not covered or only superficially addressed by the FJAS. Second, the R-Track questionnaire has previously been published in German-speaking countries regarding the job requirements of medical professionals, making such a comparison scientifically valuable. Additionally, the R-Track offers a distinct advantage over other instruments by providing a psychologically comprehensive assessment of the necessary characteristics, competencies, and skills [16].

The French-translated version used in our study includes 66 items covering the following eight competence areas: “Mental abilities” (13 questions), “Social sensibility” (5 questions), “Psychomotor and multitasking abilities” (2 questions), “Solutions orientation” (9 questions), “Social interactive competences” (14 questions), “Personality traits” (5 questions), “Verbal competences” (9 questions) and “Resistance capacity” (9 questions).

The distribution of the different items within the competence areas can be found in Supplement 2. Participants were asked to rate each item on a 5-point Likert scale (1: very low importance, 2: low importance, 3: moderate importance, 4: high importance, 5: very high importance) according to the importance attached to daily professional activity. The mean scores of each single item and of each competence domain were calculated. The ranking of single items was defined. T-tests were performed for all measures using gender and training level, and analyses of variances were calculated for place of practice.

## Results

A total of 201 physicians working in the field of obstetrics and gynecology responded resulting in a 65.5% participation rate. The mean age of participants was 42.2 ± 12.8 years. Concerning demographic data, 69.2% of participants identified themselves as female and 30.3% as male. One participant identified themself as “other” and was not included in gender comparisons, due to the non-representative nature of this category. The different levels of training were similarly represented, with 50.2% residents or fellows and 49.8% specialist physicians. The majority of participants worked in hospitals (64.7%), while 30.3% were in private practice.

Table 1. represents the ranking and mean scores of the 66 competences according to their importance for daily professional activity. “Resistance to stress” had the highest average for all participants, followed by “Workload management” and “Tactfulness”. “Resistance to monotony”, “Numeracy”, and “Mathematical reasoning” had the lowest average scores.

**Table 1:** Ranking and mean score of the R-track competencies

Item	Rank (*n*)	Mean scores and SD	Item	Rank (*n*)	Mean scores and SD	Item	Rank (*n*)	Mean scores and SD
Resistance to stress	**1**	4.66 ± 0.644	Multitasking capacity	**23**	4.00 ± 0.943	Spatial visualization	**45**	3.77 ± 1.015
Workload management	**2**	4.48 ± 0.722	Flexibility	**24**	4.00 ± 0.894	Perceptual range	**46**	3.76 ± 0.908
Tactfulness	**3**	4.44 ± 0.698	Coaching and mentoring	**25**	4.00 ± 0.935	Norms and values orientation	**47**	3.74 ± 0.913
Logical reasoning	**4**	4.44 ± 0.726	Concentration	**26**	3.99 ± 0.834	Resource awareness	**48**	3.72 ± 0.839
Coordination and decision making	**5**	4.39 ± 0.728	Willingness to help	**27**	3.98 ± 0.883	Reading comprehension	**49**	3.71 ± 0.921
Conscientiousness	**6**	4.39 ± 0.806	Verbal memory capacity	**28**	3.98 ± 0.839	Self-Reflection	**50**	3.71 ± 0.905
Partner and patient orientation	**7**	4.38 ± 0.746	Agreeableness	**29**	3.97 ± 0.911	Conflict management	**51**	3.69 ± 0.858
Emotional stability	**8**	4.36 ± 0.849	Independence and autonomy	**30**	3.92 ± 0.880	Risk tolerance		3.69 ± 0.952
Calmness	**9**	4.35 ± 0.720	Visual imagination	**31**	3.90 ± 0.897	Tolerance for frustration	**53**	3.65 ± 0.984
Understanding	**10**	4.31 ± 0.753	Authenticity	**32**	3.88 ± 0.988	Delegation	**54**	3.59 ± 0.885
Cooperation	**11**	4.29 ± 0.725	In need of harmony	**33**	3.87 ± 0.881	Presentation	**55**	3.51 ± 0.975
Endurance	**12**	4.28 ± 0.886	Role discipline	**34**	3.86 ± 0.895	Sovereignty	**56**	3.45 ± 0.910
Diplomacy	**13**	4.22 ± 0.863	Clarity of speech		3.86 ± 0.825	Persuasiveness	**57**	3.43 ± 0.876
Attention	**14**	4.20 ± 0.757	Problem solving		3.86 ± 0.861	Written expression	**58**	3.41 ± 0.966
Problem solving		4.20 ± 0.813	Considers arguments		3.86 ± 0.803	Assertiveness	**59**	3.29 ± 0.948
Rigor	**16**	4.18 ± 0.910	Retention	**38**	3.84 ± 0.780	Creativity	**60**	3.18 ± 1.014
Psychomotor coordination	**17**	4.16 ± 0.904	Hands-on	**39**	3.83 ± 0.901	Sociability	**61**	3.12 ± 0.998
Verbal comprehension	**18**	4.15 ± 0.817	Visual memory capacity	**40**	3.82 ± 0.870	Extraversion	**62**	3.04 ± 0.964
Achievement motivation	**19**	4.12 ± 0.903	Openness to novelty	**41**	3.81 ± 0.909	Facility for languages	**63**	3.01 ± 0.959
Perceptual speed	**20**	4.09 ± 0.838	Self-confidence	**42**	3.78 ± 0.814	Resistance to monotony	**64**	2.93 ± 1.134
Manners and common decency	**21**	4.05 ± 0.912	Verbal expression	**43**	3.78 ± 0.809	Numeracy	**65**	2.80 ± 0.949
Structuring information	**22**	4.04 ± 0.842	Spatial orientation	**44**	3.77 ± 0.978	Mathematical reasoning	**66**	2.71 ± 0.948

Note: Ranks are based on Mean scores only

Means and ranks of the eight competence areas according to variables can be found in Supplement 3. The analysis of the competence areas by gender, level of training, and place of practice did not reveal significant differences. The competence area “Social sensibility” received the highest ranking with a mean score of 4.17 ± 0.58, considering all participants. However, for male physicians and those working in a university hospital, “Psychomotor & multitasking abilities” was ranked highest, with mean scores of 4.20 ± 0.67 and 4.18 ± 0.73, respectively. Overall, the order of importance for the competence areas was: “Social sensibility” (4.17 ± 0.58), “Psychomotor & multitasking abilities” (4.08 ± 0.78), “Verbal competencies” (3.97 ± 0.56), “Personality traits” (3.91 ± 0.58), “Solution-oriented” (3.87 ± 0.58), “Social interactive competencies” (3.79 ± 0.54), “Mental abilities” (3.77 ± 0.51), and “Resistance capacity” (3.73 ± 0.60).

## Discussion

The acquisition of competencies is strongly dependent on existing abilities and the expression of personality traits in an individual. In this respect, the R-Track questionnaire - like other instruments - assesses both stable characteristics that are prerequisites for the acquisition of competence, as well as skills that increase the likelihood of acquisition. For example, decency is a behavior that must be learned. Nevertheless, pronounced agreeableness, a stable personality trait, is a prerequisite for a high degree of decency in social situations.

The competence areas “Social sensibility” and “Psychomotor & multitasking abilities” stand out in our sample of O&G physicians. “Resistance to stress”, “Workload management”, “Tactfulness”, “Logical reasoning”, and “Decision-making” were considered the most important skills for the daily professional activity. With no significant differences according to gender, level of training, or place of practice, these results can be generalized to the entire sample.

The identified competence profile could serve as a basis for developing a new method of personalized residency selection for O&G and improve recruitment-based factors other than internship reports, letters of recommendation, medical school grades, or personal interviews. Indeed, personalizing a selection process and focusing on skills deemed important in this medical specialty is essential to reflect the values and needs of this specific program [5].

On the other hand, these results may also enable candidates to compare their personal competence profile with that of the field and assess its fit, data to the few available [11, 17–18].

Testing “Social sensibility” and “Psychomotor & multitasking abilities” among students applying for an O&G residency program requires innovative evaluation methods. Strategies that residency programs may use to assess these qualities include simulated patient encounters, where applicants’ communication skills, empathy, and ability to navigate complex social dynamics while providing patient-centered care are tested. Objective Structured Clinical Examinations (OSCEs) are structured assessments that evaluate these clinical skills, communication, and professionalism in a standardized manner [19].

Another method of evaluation could be the situational Judgment Tests, where applicants evaluate behavioral responses in context-relevant situations. This validated approach shows promise in assessing noncognitive attributes in residency program candidates [20, 21].

Regarding multitasking and psychomotor abilities, we could consider using a simulated multitasking scenario that incorporates various skills important in the medical field, each with different levels of priority and difficulty. In this scenario, participants perform these tasks simultaneously, assigning priorities based on their judgment [22, 23]. For example, applicants could be presented with simulated scenarios involving labor and delivery, requiring them to manage multiple tasks simultaneously, such as monitoring fetal heart rate, communicating with the patient and family, and coordinating care with other healthcare providers.

Another approach for testing multitasking ability could be the use of the Multi-Attribute Task Battery, a computerized flight simulator designed for aviation tasks, suitable for both pilots and non-pilots. It involves the execution, either individually or simultaneously, of four sub-tasks: system monitoring, tracking, communication, and resource management [24].

By incorporating these assessment methods into the selection process, residency programs can gain a more holistic understanding of applicants’ social sensibility and psychomotor/multitasking abilities.

Among the available data related to personality traits in the different medical specialties, we would highlight the literature review by Borges et al. In their work, obstetricians and gynecologists are described as sensitive, conscientious, organized, persistent, scrupulous, and achievement-oriented. These character traits observed in O&G providers are found in the top third of skills deemed necessary for the practice of the profession in our sample. Only the extroverted nature of obstetricians and gynecologists described in Borges’ work, was not considered an important characteristic of our sample [25].

Our results align with those of Zelesniack et al. who defined the competence profile of different medical activities. As for other specialties with a surgical component, the competence area “Psychomotor & multitasking abilities” was judged very important. The competence areas “Personality traits” and “Social interactive” were considered less important in our sample, in contrast to non-surgical specialties such as psychiatry, internal medicine, or pediatrics, for which these competence areas are among the most important [11].

Another point worth mentioning is that the characteristics “Resistance to stress” and “Workload management” ranked as the two most important competences in our sample. This result raises the important issue of high stress and workload among physicians. Burnout among this group of professionals has already been the subject of numerous studies [26]. O&G providers face significant risk of burnout due to the demanding and emotionally challenging nature of their work [27–28]. Indeed, they deal with high-stakes situations such as labor and delivery, and managing complex gynecological conditions and surgeries. The unpredictable nature of obstetric emergencies and the need to make critical decisions quickly can lead to chronic stress. Overnight and weekend shifts can disrupt work-life balance and contribute to fatigue and burnout. They also frequently encounter emotionally charged situations, including pregnancy complications, fetal or neonatal deaths, infertility, miscarriages, and gynecological cancers. Finally, O&G providers are at the highest risk of medical malpractice lawsuits among all medical specialties, given the nature of their specialty and the potential for adverse outcomes in obstetrics and gynecology [29]. Fear of litigation and the need to practice defensive medicine can increase stress and anxiety.

We believe that focusing on the identification of specific competencies for resident recruitment is crucial for creating a selection process that aligns candidates’ natural skills with the actual demands of the O&G specialty. This approach ultimately enhances training effectiveness, reduces the risk of burnout, and supports long-term professional fulfillment, which benefits both doctors and patients.

Adding data to the almost non-existent literature on this topic is the main strength of our study. With a participation rate of 65%, our sample of 201 participants is highly satisfactory. The homogeneity between training levels and practice locations is another strength.

As a weakness, we will note the local nature of this sample, concentrated solely in French-speaking Switzerland, for which the results can only be generalized with caution. Indeed, the day-to-day activities of this branch can vary from region to region, in terms of workload, working hours, available resources and legal protection. We can imagine carrying out the same analyses in other regions where the healthcare system differs, in order to investigate whether this has an impact on the skills profile required. Regarding other biases, the mode of distribution (e.g., email used in our study vs. physical mail) can affect response rates. For example, older doctors might prefer paper forms, while younger doctors might be more likely to respond to an online questionnaire, leading to a potential age-related bias. However, emails are used by all doctors in the French-speaking part of Switzerland, as all patient charts are now exchanged through a secure process using email.

Finally, in today’s patient-centered approach to medicine, our study’s analysis should also consider the perspectives of patients in our region regarding what they value most. Selecting doctors who align with patients’ needs is essential for providing optimal care.

## Conclusion

Among physicians working in O&G, the competence areas “Social sensibility” and “Psychomotor & multitasking abilities” are considered to be the most important for daily professional activity. “Resistance to stress”, “Workload management” and “Tactfulness” are the characteristics judged to be the most essential. The identified competence profile could serve as a basis for developing a new method of residency selection in obstetrics and gynecology.

## Electronic supplementary material

Below is the link to the electronic supplementary material.

Supplementary Material 1

## Data Availability

The datasets analyzed during the current study are available from the corresponding author upon reasonable request.

## References

[1] Tidwell J, Yudien M, Rutledge H, Terhune KP, LaFemina J, Aarons CB. Reshaping Residency Recruitment: achieving alignment between applicants and programs in surgery. J Surg Educ. 2022;79(3):643–54. 35123913 10.1016/j.jsurg.2022.01.004

[2] Vaughan LA, Quick JA. Evidence-based selection of Surgical residents. Surg Clin North Am. 2021;101(4):667–77. 34242608 10.1016/j.suc.2021.05.012

[3] Kenny S, McInnes M, Singh V. Associations between residency selection strategies and doctor performance: a meta-analysis. Med Educ. 2013;47(8):790–800. 23837425 10.1111/medu.12234

[4] Schaverien MV. Selection for Surgical training: an evidence-based review. J Surg Educ. 2016;73(4):721–9. 27133583 10.1016/j.jsurg.2016.02.007

[5] Naides AI, Ayyala HS, Lee ES. How do we choose? A review of Residency Application Scoring systems. J Surg Educ. 2021;78(5):1461–8. 33744118 10.1016/j.jsurg.2021.02.003

[6] Bell JG, Kanellitsas I, Shaffer L. Selection of obstetrics and gynecology residents on the basis of medical school performance. Am J Obstet Gynecol. 2002;186(5):1091–4. 12015542 10.1067/mob.2002.121622

[7] Patel H, Yakkanti R, Bellam K, Agyeman K, Aiyer A. Innovation in Resident Selection: life without step 1. J Med Educ Curric Dev. 2022;9:23821205221084936. 35372695 10.1177/23821205221084936 PMC8968982

[8] Stohl HE, Hueppchen NA, Bienstock JL. Can medical school performance predict residency performance? Resident selection and predictors of successful performance in obstetrics and gynecology. J Grad Med Educ. 2010;2(3):322–6. 21976076 10.4300/JGME-D-09-00101.1 PMC2951767

[9] EASA [Internet]. 2017 [cited 2024 Mar 8]. CRM Training Implementation. Available from: https://www.easa.europa.eu/en/document-library/general-publications/crm-training-implementation

[10] Fürstenberg S, Schick K, Deppermann J, Prediger S, Berberat PO, Kadmon M, et al. Competencies for first year residents - physicians’ views from medical schools with different undergraduate curricula. BMC Med Educ. 2017;17(1):154. 28882189 10.1186/s12909-017-0998-9 PMC5590189

[11] Zelesniack E, Oubaid V, Harendza S. Defining competence profiles of different medical specialties with the requirement-tracking questionnaire - a pilot study to provide a framework for medial students’ choice of postgraduate training. BMC Med Educ. 2021;21(1):46. 33435986 10.1186/s12909-020-02479-6 PMC7801870

[12] Bexelius TS, Olsson C, Järnbert-Pettersson H, Parmskog M, Ponzer S, Dahlin M. Association between personality traits and future choice of specialisation among Swedish doctors: a cross-sectional study. Postgrad Med J. 2016;92(1090):441–6. 26864310 10.1136/postgradmedj-2015-133478

[13] Mullola S, Hakulinen C, Presseau J, Gimeno Ruiz de Porras D, Jokela M, Hintsa T, et al. Personality traits and career choices among physicians in Finland: employment sector, clinical patient contact, specialty and change of specialty. BMC Med Educ. 2018;18:52. 29587722 10.1186/s12909-018-1155-9 PMC5870817

[14] Xiao Y, Dong M, Shi C, Zeng W, Shao Z, Xie H, et al. Person-environment fit and medical professionals’ job satisfaction, turnover intention, and professional efficacy: a cross-sectional study in Shanghai. PLoS ONE. 2021;16(4):e0250693. 33905430 10.1371/journal.pone.0250693 PMC8078800

[15] Fleishman E, Reilly M. Fleishman Job Analysis Survey. Administrator guide. Potomac: MD Management Research Institute; 1995.

[16] Oubaid V. Der Faktor Mensch. Berlin: MWV-; 2019.

[17] Jebram L, Prediger S, Oubaid V, Harendza S. Matching of advanced undergraduate medical students’ competence profiles with the required competence profiles of their specialty of choice for postgraduate training. BMC Med Educ. 2023;23(1):647. 37679688 10.1186/s12909-023-04632-3 PMC10485971

[18] Zelesniack E, Oubaid V, Harendza S. Final-year medical students’ competence profiles according to the modified requirement tracking questionnaire. BMC Med Educ. 2021;21(1):319. 34088296 10.1186/s12909-021-02728-2 PMC8178874

[19] Khan KZ, Ramachandran S, Gaunt K, Pushkar P. The Objective Structured Clinical Examination (OSCE): AMEE Guide 81. Part I: an historical and theoretical perspective. Med Teach. 2013;35(9):e1437–1446. 23968323 10.3109/0142159X.2013.818634

[20] Mielke I, Breil SM, Amelung D, Espe L, Knorr M. Assessing distinguishable social skills in medical admission: does construct-driven development solve validity issues of situational judgment tests? BMC Med Educ. 2022;22(1):293. 35440029 10.1186/s12909-022-03305-x PMC9020047

[21] Cullen MJ, Zhang C, Marcus-Blank B, Braman JP, Tiryaki E, Konia M, et al. Improving our ability to Predict Resident Applicant performance: Validity evidence for a situational Judgment Test. Teach Learn Med. 2020;32(5):508–21. 32427496 10.1080/10401334.2020.1760104

[22] Adams TN, Rho JC. Multitasking simulation: Present application and future directions. Med Teach. 2017;39(2):120–2. 27633071 10.1080/0142159X.2016.1230666

[23] Huang WC, Hsu SC, Yang CH, Lin CW, Suk FM, Hu KC, et al. A novel approach: simulating multiple simultaneous encounters to assess multitasking ability in emergency medicine. PLoS ONE. 2021;16(9):e0257887. 34582505 10.1371/journal.pone.0257887 PMC8478191

[24] Pontiggia A, Gomez-Merino D, Quiquempoix M, Beauchamps V, Boffet A, Fabries P, et al. MATB for assessing different mental workload levels. Front Physiol. 2024;15:1408242. 39108543 10.3389/fphys.2024.1408242 PMC11300324

[25] Borges N, Savickas M. Personality and Medical Specialty Choice: A literature review and integration. J Career Assess - J CAREER Assess. 2002;10:362–80.

[26] Rotenstein LS, Torre M, Ramos MA, Rosales RC, Guille C, Sen S, et al. Prevalence of Burnout among Physicians: a systematic review. JAMA. 2018;320(11):1131–50. 30326495 10.1001/jama.2018.12777 PMC6233645

[27] Smith RP. Burnout in obstetricians and gynecologists. Clin Obstet Gynecol. 2019;62(3):405–12. 30921003 10.1097/GRF.0000000000000441

[28] Smith RP, Rayburn WF. Burnout in Obstetricians-Gynecologists: its Prevalence, Identification, Prevention, and reversal. Obstet Gynecol Clin North Am. 2021;48(1):231–45. 33573788 10.1016/j.ogc.2020.11.008

[29] Ghaith S, Campbell RL, Pollock JR, Torbenson VE, Lindor RA. Medical malpractice lawsuits involving trainees in Obstetrics and Gynecology in the USA. Healthc Basel Switz. 2022;10(7):1328. 10.3390/healthcare10071328 PMC9319230 35885853

