# Supplementary material for: Defining competence profiles in obstetrics and gynecology using the modified requirement tracking questionnaire
Source: BMC Med Educ. 2025 Feb 7;25:204. doi: 10.1186/s12909-025-06806-7 (PMC11806616; doi:10.1186/s12909-025-06806-7)
Supplement: Supplementary file 1 — Supplementary Material 1 [file 12909_2025_6806_MOESM1_ESM.docx]

**Supplement 1. R-Track questionnaire translated from French to English**

**Socio-demographic characteristics**

**1.**

**Type of clinic you work at**

University hospital (hôpital universitaire)

Regional clinic (hôpital de périphérie)

Doctor's practice (cabinet)

**2.**

**Please indicate your current position**

Head physician

Senior physician

Assistant Doctor in 3rd year and +

Assistant Doctor in 1st & 2nd year

Student assistentship

**3.**

**Experience in your current position (in years)**

**4.**

**Overall job experience (in years)**

**5.**

**Age (in years)**

**6.**

**Gender**

female

male

diverse

**R-track items to be rated from 1 to 5 according to the following instruction:**

Please rate on a scale of 1 to 5 the importance of the following elements for your daily professional activity.

A mark of 1 indicates "low importance", i.e. the feature is relatively unimportant for the job.

A mark of 2 indicates "low importance".

A mark of 3 corresponds to "medium importance": the characteristic is not unimportant, but is no more important than for medical fields other than gynecology/obstetrics.

A score of 4 is equivalent to "rather high importance".

A score of 5 is equivalent to "high importance"; the characteristic is imperative for the position

**1.**

**Coordination and decision making**

Can lead and make decisions

**2.**

**Risk tolerance**

Is daring and willing to take risks

**3.**

**Diplomacy**

Is verbally skilled and can act considerately

**4.**

**Emotional stability**

Can hold back emotionally and control him/herself

**5.**

**Workload management**

Recognizes situational demands and is able to make space for performance

**6.**

**Extraversion**

Tendency to enjoy human interactions and to be enthusiastic, talkative, assertive, and gregarious.

**7.**

**Conscientiousness**

Being careful or diligent. Conscientiousness implies a desire to do a task well, and to take obligations to others seriously.

**8.**

**Agreeableness**

Being kind, sympathetic, cooperative, warm, and considerate

**9.**

**Persuasiveness**

Defends his/her points of view in convincing fashion

**10.**

**Tactfulness**

Shows empathy and sensitivity towards others

**11.**

**Creativity**

Solves problems in a creative and ingenious manner

**12.**

**Openness to novelty**

Is open to new experiences, mentalities and cultures

**13.**

**Flexibility**

Can adapt, does not insist on familiarity

**14.**

**Self-reflection**

Can evaluate and describe him/herself in differentiated manner

**15.**

**Independence and autonomy**

Acts autonomously without instruction or control

**16.**

**Problem solving**

Can implement ideas effectively

**17.**

**Assertiveness**

Deliberately asserts his/herself

**18.**

**Calmness**

Does not act prematurely, calm and collected in action

**19.**

**Thoroughness**

Knows rules and follows them in disciplined fashion

**20.**

**Resistance to stress**

Handles stressful situations appropriately

**21.**

**Resistance to monotony**

Tolerates breaks and waiting periods

**22.**

**Tolerance for frustration**

Tolerates problems and setbacks and processes them constructively

**23.**

**Achievement motivation**

Tackles essential tasks with energy and stamina until successful completion

**24.**

**Verbal expression**

Can express him/herself adequately and eloquently

**25.**

**Presentation**

Presents him/herself confidently and convincingly

**26.**

**Conflict management**

Recognizes and addresses conflicts

**27.**

**Cooperation**

Is cooperative when dealing with others

**28.**

**Willingness to help**

Helps and supports, even to the detriment of own goals

**29.**

**In need of harmony**

Strives for harmony within teams

**30.**

**Delegation**

Can delegate responsibilities and tasks to colleagues

**31.**

**Structuring Information**

Can meaningfully separate and arrange information

**32.**

**Self-confidence**

Shows confident attitude and defends his/her positions

**33.**

**Sociability**

Is extroverted and likes to surround him/herself with people

**34.**

**Written expression**

Writes clearly and makes correct use of grammar

**35.**

**Manners and common decency**

Follows social conventions and behaves adequately, also in multicultural environments

**36.**

**Clarity of speech**

Speaks clearly and asks questions efficiently

**37.**

**Hands-On**

Adopts a “hands-on” approach, irrespectively of hierarchy

**38.**

**Considers arguments**

Processes arguments of others in constructive and discursive fashion

**39.**

**Role discipline**

Stays in his/her role, minds compliance with role distribution

**40.**

**Norms and values orientation**

Knows norms and values, attaches great importance to their adherence from self and others

**41.**

**Authenticity**

Is authentic, genuine and does not pretend

**42.**

**Coaching and mentoring**

Advises, supports, and shares knowledge with others, leads the way

**43.**

**Patients orientation**

Is oriented towards patients satisfaction

**44.**

**Resource awareness**

Mobilizes personal and material resources very sparingly

**45.**

**Sovereignty**

Appears and acts confidently

**46.**

**Endurance**

Shows personal fitness

**47.**

**Logical reasoning**

Understands problems and thinks in task-oriented fashion

**48.**

**Numeracy**

Calculates quickly and accurately

**49.**

**Verbal memory capacity**

Can easily remember verbal information

**50.**

**Visual memory capacity**

Can easily remember visual information

**51.**

**Retention**

Displays adequate visual and auditory memory capacity

**52.**

**Perceptual speed**

Quickly registers external stimulation

**53.**

**Understanding**

Accurately differentiates information

**54.**

**Perceptual range**

Utilizes different information and perceptual channels simultaneously

**55.**

**Visual imagination**

Can imagine things visually

**56.**

**Facility for languages**

Understands languages and accents effortlessly

**57.**

**Reading comprehension**

Understands writing effortlessly

**58.**

**Problem comprehension**

Understands logical connections

**59.**

**Verbal understanding**

Understands spoken language effortlessly

**60.**

**Attention**

Can effortlessly focus perception and action on relevant tasks

**61.**

**Psychomotor coordination**

Effortless hand-eye-coordination, accurate movements, finger dexterity

**62.**

**Multitasking capacity**

Performs more than one task, or activity, at the same time, including psychomotor activity

**63.**

**Mathematical reasoning**

Is able to reflect on mathematical / numerical solutions to problems and determine whether or not they make sense

**64.**

**Concentration**

Focus attention one task for a longer time

**65.**

**Spatial orientation**

Is able to perceive and adjust one's location in space in relation to objects in the external environment

66.

**Spatial visualization**

Is able to mentally manipulate 2-dimensional and 3-dimensional figures

**Supplement 2. Distribution of the different items within the competence areas**

**Mental abilities (13 questions)**

- Logical reasoning
- Numeracy
- Verbal memory capacity
- Visual memory capacity
- Retention
- Perceptual speed
- Perceptual range
- Visual imagination
- Attention
- Mathematical reasoning
- Concentration
- Spatial orientation
- Spatial visualization

**Social sensibility (5 questions)**

- Tactfulness
- Willingness to help
- Manners and common decency
- Coaching and mentoring
- Attention to patients/partner

**Psychomotor and multitasking abilities (2 questions)**

- Psychomotor coordination
- Multitasking capacity

**Solutions orientation (9 questions)**

- Creativity
- Flexibility
- Independence and autonomy
- Problems solving
- Thoroughness
- Achievement motivation
- Structuring Information
- Hands-On
- Resource awareness

**Social interactive competences (14 questions)**

- Diplomacy
- Persuasiveness
- Self-reflection
- Calmness
- Presentation
- Cooperation
- In need of harmony
- Delegation
- Sociability
- Written expression
- Clarity of speech
- Considers arguments
- Norms and values orientation
- Authenticity

**Personality traits (5 questions)**

- Emotional stability
- Extraversion
- Conscientiousness
- Agreableness
- Openness to novelty

**Verbal competences (9 questions)**

- Verbal expression
- Conflict management
- Clarity of speech
- Understanding
- Facility for languages
- Reading comprehension
- Problem comprehension
- Information structure
- Verbal understanding

**Resistance capacity (9 questions)**

- Assertiveness
- Resistance to stress
- Resistance to monotony
- Tolerance for frustration
- Conflict management
- Self-confidence
- Role discipline
- Sovereignty
- Endurance

**Supplement 3. Means and ranks of the eight competence areas according to variables**

|  | **Competence areas** | | | | | | | |
| --- | --- | --- | --- | --- | --- | --- | --- | --- |
| **Variables** | **Mental abilities MW ± SD (rank)** | **Social sensibility MW ± SD (rank)** | **Psychomotor & multitasking abilities MW ± SD (rank)** | **Solution-oriented MW ± SD (rank)** | **Social interactive competencies MW ± SD (rank)** | **Personality traits MW ± SD (rank)** | **Verbal competencies MW ± SD (rank)** | **Resistance capacity MW ± SD (rank)** |
| **Total** (n=201) | 3.77 ± 0.513 (7) | 4.17 ± 0.583 (1) | 4.08 ± 0.784 (2) | 3.87 ± 0.575 (5) | 3.79 ± 0.543 (6) | 3.91 ± 0.576 (4) | 3.97 ± 0.556 (3) | 3.73 ± 0.599 (8) |
| **Gender** |  |  |  |  |  |  |  |  |
| **Female** (n=139) | 3.74 ± 0.563 (7) | 4.17 ± 0.487 (1) | 4.04 ± 0.825 (2) | 3.88 ± 0.611 (5) | 3.78 ± 0.576 (6) | 3.90 ± 0.614 (4) | 3.97 ± 0.583 (3) | 3.71 ± 0.640 (8) |
| **Male** (n=61) | 3.86 ± 0.366 (5) | 4.17 ± 0.622 (2) | 4.20 ± 0.667 (1) | 3.86 ± 0.486 (5) | 3.82 ± 0.468 (7) | 3.92 ± 0.474 (4) | 3.96 ± 0.496 (3) | 3.80 ± 0.493 (8) |
| **Level of training** |  |  |  |  |  |  |  |  |
| **In training** (n=101) | 3.70 ± 0.491 (7) | 4.16 ± 0.531 (1) | 4.06 ± 0.747 (2) | 3.80 ± 0.538 (5) | 3.77 ± 0.524 (6) | 3.86 ± 0.553 (4) | 3.91 ± 0.539 (3) | 3.65 ± 0.540 (8) |
| **Trained** (n=100) | 3.85 ± 0.527 (6) | 4.17 ± 0.634 (1) | 4.10 ± 0.823 (2) | 3.95 ± 0.603 (4) | 3.82 ± 0.564 (7) | 3.95 ± 0.598 (4) | 4.02 ± 0.570 (3) | 3.82 ± 0.644 (7) |
| **Place of practice** |  |  |  |  |  |  |  |  |
| **University hospital** (n=66) | 3.80 ± 0.414 (7) | 4.16 ± 0.518 (2) | 4.18 ± 0.732 (1) | 3.89 ± 0.523 (5) | 3.84 ± 0.546 (6) | 3.92 ± 0.532 (4) | 3.95 ± 0.511 (3) | 3.70 ± 0.512 (8) |
| **Regional hospital** (n=64) | 3.70 ± 0.551 (7) | 4.22 ± 0.488 (1) | 4.05 ± 0.744 (2) | 3.77 ± 0.518 (5) | 3.73 ± 0.454 (6) | 3.89 ± 0.521(4) | 3.93 ± 0.503 (3) | 3.67 ± 0.532 (8) |
| **Private practice** (n=71) | 3.81 ± 0.559 (7) | 4.13 ± 0.710 (1) | 4.02 ± 0.864 (2) | 3.95 ± 0.659 (4) | 3.81 ± 0.613 (7) | 3.93 ± 0.664 (5) | 4.01 ± 0.639 (3) | 3.82 ± 0.718 (6) |
